# Validation of a one-tube nested real-time PCR assay for the detection of *Cryptosporidium* spp. in avian fecal samples

**DOI:** 10.1590/S1984-29612022017

**Published:** 2022-03-21

**Authors:** Bruna Nicoleti Santana, Elis Domingos Ferrari, Alex Akira Nakamura, Giane Serafim da Silva, Marcelo Vasconcelos Meireles

**Affiliations:** 1 Faculdade de Medicina Veterinária, Universidade Estadual Paulista – UNESP, Araçatuba, SP, Brasil; 2 Laboratório de Parasitologia Animal, Instituto Biológico, Votuporanga, SP, Brasil

**Keywords:** Cryptosporidiosis, diagnosis, polymerase chain reaction, Criptosporidiose, diagnóstico, reação em cadeia pela polimerase

## Abstract

The aim of this study was to validate a one-tube nested real-time PCR assay followed by genetic sequencing to detect and identify *Cryptosporidium* species and genotypes in birds. A total of 443 genomic DNA extracted from avian fecal samples were analyzed by one-tube nested real-time PCR and conventional nested PCR. By one-tube nested real-time PCR, 90/443 (20.3%) samples were positive for *Cryptosporidium* spp. In contrast, 36/443 (8.1%) samples were positive for *Cryptosporidium* spp. by conventional nested PCR. The analytical sensitivity test showed that one-tube nested real-time PCR detects approximately 0.5 oocyst (2 sporozoites) per reaction. An evaluation of analytical specificity did not reveal amplification of microorganisms that commonly present nonspecific amplification with primers used for the diagnosis of *Cryptosporidium* spp. The repeatability analysis showed the same result in 27 out of 30 samples (90%). As for the reproducibility of one-tube nested real-time PCR, 24 of the 30 samples examined (80%) showed the same result. All the 90 samples amplified by one-tube real-time nested PCR were successfully sequenced, leading to the identification of *C. baileyi, C. galli, C. meleagridis, C. proventriculi*, and *Cryptosporidium* avian genotype I. Genetic sequencing of conventional nested PCR amplicons was successful in 10/36 (27.8%) of positive samples.

## Resumo

O objetivo deste trabalho foi validar um protocolo de *nested* PCR em tempo real em um tubo (nPCR-TR-1T) seguida de sequenciamento genético para detectar e caracterizar as espécies e genótipos de *Cryptosporidium* em aves. Um total de 443 amostras de DNA genômico, extraído de amostras fecais de aves, foi analisado pela nPCR-TR-1T e pela *nested* PCR convencional. Pela nPCR-TR-1T, foi observada positividade para *Cryptosporidium* spp. de 20,3% (90/443), em contraste com a *nested* PCR convencional, que apresentou positividade de 8,1% (36/443). O teste de sensibilidade analítica mostrou que a nPCR-TR-1T detecta aproximadamente 0,5 oocisto (2 esporozoítos) por reação. A avaliação da especificidade analítica não revelou amplificação de microrganismos que comumente apresentam amplificação inespecífica com *primers* utilizados para o diagnóstico de *Cryptosporidium* spp. O cálculo da repetibilidade evidenciou o mesmo resultado em 27 de 30 amostras (90%). Em relação à reprodutibilidade da nPCR-TR-1T, foi observado o mesmo resultado em 80% (24/30) das amostras examinadas. Foi possível realizar o sequenciamento em todas as 90 amostras amplificadas pela nPCR-TR-1T, com identificação de *C. baileyi, C. galli, C. meleagridis, C. proventriculi* e *Cryptosporidium* genótipo I em aves. O sequenciamento dos fragmentos amplificados pela *nested* PCR convencional foi possível em 10/36 (27,8%) das amostras positivas.

## Introduction

Cryptosporidiosis is one of the main protozoan infections in birds, presenting as an acute disease in the respiratory or digestive tracts of several avian species, including domestic chickens, quails, turkeys, pheasants, peacocks, and other domestic and wild bird species (Sréter & Varga, 2000; Nakamura & Meireles, 2015).

Six *Cryptosporidium* species infect birds, namely, *C. meleagridis* (Slavin, 1955), *C. baileyi* (Current et al., 1986), *C. galli* (Ryan et al., 2003), *C. avium* (avian genotype V) (Holubová et al., 2016), *C. proventriculi* (avian genotype III) (Abe & Makino, 2010; Holubová et al., 2019), and *C. ornithophilus* (avian genotype II) (Santos et al., 2005; Meireles et al., 2006; Ng et al., 2006; Holubová et al., 2020). *Cryptosporidium* avian genotypes I, IV, VI, VII, VIII, and IX (Ng et al., 2006; Helmy et al., 2017) and *Cryptosporidium xiaoi*-like genotype (Ewald et al., 2017; Santana et al., 2018; Liu et al. 2019; Gong et al., 2021) have also been described in birds.

Studies carried out in Australia, China and Nigeria using fecal samples from several bird species have reported *Cryptosporidium* spp. infection rates of 4.9% to 8.1%, in addition to the detection of *Cryptosporidium andersoni*, *C. avium, C. baileyi,* and the avian genotypes I and II (Ng et al., 2006; Qi et al., 2011; Bamaiyi et al., 2013).

Infections by *C. baileyi*, *C. galli, C. proventriculi, C. avium, C. ornithophilus*, and by the various *Cryptosporidium* genotypes are found in a wide variety of bird species, while *C. meleagridis* has a more restricted number of avian hosts (Sréter & Varga, 2000; Ng et al., 2006; Nakamura et al., 2009; Qi et al., 2011, Holubová et al., 2019; Holubová et al., 2020).

Gastrointestinal infection by *C. meleagridis, C. galli* and *C. proventriculi* may result in clinical signs ranging from anorexia to diarrhea, weight loss and chronic vomiting (Sréter & Varga, 2000; Makino et al., 2010; Ravich et al., 2014). Infection by *C. baileyi* is considered part of the respiratory complex of birds, as a primary or secondary disease (Blagburn et al., 1987). In respiratory infections caused by *C. baileyi*, mucus accumulates in the trachea, lungs, and air sacs, with clinical signs characterized by dyspnea, coryza and sometimes death (Nakamura & Meireles, 2015). The importance of *Cryptosporidium* spp. in the intestine and/or bursa of Fabricius of most avian species remains undefined, although intestinal infection can result in clinical disease and increased mortality in turkeys and quails (Hoerr et al., 1986) and infection in the bursa of Fabricius can result in immunosuppression (Naciri et al., 1989; Eladl et al., 2014). As for *C. parvum*, there is a report of natural infection associated to enteritis in Stone curlews (*Burhinus oedicnemus*) (Zylan et al., 2008).

The technique universally employed for the diagnosis and genotypic characterization of *Cryptosporidium* spp. in avian fecal samples is conventional nested PCR to amplify a fragment of the 18S rRNA gene followed by genetic sequencing (Xiao et al., 1999; Xiao et al., 2000; Nakamura & Meireles, 2015). Although this technique is used in most cryptosporidiosis research and diagnostic laboratories, conventional nested PCR is time-consuming because it is performed in two steps and requires electrophoresis to visualize the amplified fragment. This means the diagnosis takes longer, the probability of contamination of the laboratory environment with amplicons resulting from the two reactions (PCR and nested PCR) is greater, and the cost of consumables is higher.

Real-time PCR is an alternative method to conventional PCR, providing a faster diagnosis and reducing possible contamination in the laboratory. In addition, it does not require electrophoresis to reveal the results, preliminary results may be examined before the end of the reaction, and the degree of infection can be determined based on the absolute quantification of the amplified DNA (Arya et al., 2005).

Most real-time PCR protocols currently available for the detection of *Cryptosporidium*, which are employed to analyze samples from different species of animals and humans, are species-specific (Homem et al., 2012; Staggs et al., 2013; Yang et al., 2013; Silva et al., 2014; Nakamura et al., 2014). A real-time PCR protocol was developed for the genus-specific diagnosis of *Cryptosporidium* in human fecal samples, with amplification of a ~300 bp fragment of the 18S rRNA gene and genetic sequencing for the identification of *Cryptosporidium* species (Hadfield et al., 2011). However, in our laboratory, this protocol resulted in nonspecific amplification in samples from several animal species.

For the species-specific diagnosis of *Cryptosporidium*, the sequence of fragments amplified by PCR must contain conserved regions for annealing of genus-specific primers and internal polymorphic regions that enable differentiation of different species and genotypes by genetic sequencing. The recommended amplicon size to perform real-time PCR assays is, at most, 300 bp (Arya et al., 2005). This is probably why all the protocols for the detection and identification of *Cryptosporidium* spp. are related to conventional PCR or nested PCR followed by genetic sequencing of a partial fragment of the 18S rRNA gene. This gene is universally used for this purpose due to its composition with highly conserved nucleotide sequences for the entire genus *Cryptosporidium* interspersed with polymorphic regions, which enable the identification of *Cryptosporidium* species and genotypes by genetic sequencing (Xiao et al., 1999). Most regions of this gene that have these characteristics are equivalent to fragments larger than 300 bp.

An alternative to nested PCR (conventional or real-time), commonly performed in two steps and in two tubes, is to perform two steps in a single tube. One-tube real-time PCR has similar sensitivity to PCR protocols performed in two tubes for the detection of DNA from bacteria (Choi et al., 2014), fungi (Thongsri et al., 2013), viruses (Atkinson et al., 2014) and plants (Costa et al., 2013). With regard to cryptosporidiosis, Minarovicová et al. (2009) developed a method using one-tube nested real-time PCR for the specific the detection of *C. parvum* using a species-specific probe.

There are no studies focusing on the application of one-tube nested real-time PCR protocols for the detection of *Cryptosporidium* spp. in birds. Therefore, the validation of an assay for this purpose would provide an alternative to currently available methods, with high sensitivity, high specificity, lower consumption of reagents, less possibility of contamination and faster results. Thus, the objectives of this study were to validate a one-tube nested real-time PCR assay protocol for the detection of *Cryptosporidium* spp. in avian fecal samples and the subsequent identification of the *Cryptosporidium* species and genotypes by genetic sequencing.

## Material and Methods

This study was carried out after approval by the Ethics Committee on Animal Use of the Faculty of Veterinary Medicine of UNESP (São Paulo State University) at Araçatuba (FOA Process nos. 280-2018 and 790-2019).

The steps for validation of real-time PCR protocols recommended by Toohey-Kurth et al. (2020) were performed in the following sequence: feasibility studies, development and standardization of the reaction, and determination of analytical sensitivity and specificity, diagnostic sensitivity and specificity, repeatability, and reproducibility.

### Nested one-tube real-time PCR for amplification of 18S rRNA gene fragment

Sequences of PCR primers were defined using the Primer Blast tool with the addition of a sequence of 15 and 14 nucleotides at the 5’ end of CPB-DIAGF and CPB-DIAGR primers, respectively (Table 1), in order to allow the annealing of the primers at a temperature of approximately 10°C above the annealing temperature of the nested PCR primers (Minarovičová et al., 2009; Atkinson et al., 2014; Sun et al., 2017; Shen et al., 2018). Nested PCR primers CPB-DIAGF and CPB-DIAGR (Table 1) were previously developed by Johnson et al. (1995) to perform a conventional nested PCR protocol for the detection of *Cryptosporidium* spp.

**Table 1:** Primers used in one-tube nested real-time PCR targeting the 18S rRNA gene of *Cryptosporidium* spp.

***Primers***	**Position** *	**5´- 3´ sequences**	**Amplified product (bp)**	**Reference**
NRT18SF	584-616	GTTGTTGCAGTTAAAAAGCTCGTAGTTGGATT	~457	This study
NRT18SR	1010-1040	ACTTTGATTTCTCATAAGGTGCTGAAGGAGT		
CPB-DIAGF	599-619	AAGCTCGTAGTTGGATTTCTG	~428	Johnson et al. (1995)
CPB-DIAGR	1006-1026	TAAGGTGCTGAAGGAGTAAGG		

* Position on *C. baileyi* 18S rRNA gene (*GenBank* accession number AF093495).

Tests were performed to standardize the two steps of one-tube nested real-time PCR. Each reaction consisted of a total volume of 20 μl, with 10 μl of SsoFast® EvaGreen Supermix (Bio-Rad), 2 microliters of target DNA (*C. parvum* and *Cryptosporidium serpentis* genomic DNA), 10 to 100 nM of primers (PCR) and 100 to 500 nM of primers (nested PCR), and ultrapure water. The reactions were performed in a CFX96™ Real-Time PCR Detection System (Bio-Rad), using real-time PCR microplates (Axygen). Fluorescence emission was read in the nested PCR step. Tests were also performed by varying the primer annealing temperature and extension time and the number of cycles in each step. The number of cycles analyzed in the PCR and nested PCR steps was 10 to 20 and 30 to 40, respectively.

The dissociation temperature of the one-tube nested real-time PCR amplicon was determined by melting curve analysis, at temperatures ranging from 65°C to 95°C, with 0.5°C increments and reading for 5s. One-tube nested real-time PCR amplicons were subjected to agarose gel electrophoresis to confirm the size of the amplified fragment and verify the presence of nonspecific amplification.

The choice of the best conditions for the one-tube nested real-time PCR was based on the evaluation of the amplification curve (exponential, linear and plateau phases) and observation of the presence of peaks suggestive of primer dimers, in the analysis of the dissociation curve.

After the standardization step, one-tube nested real-time PCR was performed using a volume of 20 µl, with 10 µl of SsoFast™ EvaGreen Supermix (Bio-Rad), 0.6 µg/µl of non-acetylated bovine serum albumin (Sigma-Aldrich), 50 nM and 400 nM of each primer in the 1^st^ and 2^nd^ steps, respectively, plus 2 µl of target DNA and ultrapure water. The reactions were performed in a CFX96™ Real-Time PCR Detection System (Bio-Rad), in 0.1 ml real-time PCR microtubes (Scientific Specialties). Fluorescence emission was read in the nested PCR step. The PCR step consisted of initial denaturation at 98º C for two minutes, followed by 20 cycles of denaturation at 98º C for five seconds, and simultaneous annealing and extension at 70º C for 30 seconds. The 2^nd^ amplification step consisted of 35 cycles of 98º C for five seconds, annealing at 62º C for 30 seconds and extension at 72º C for 20 seconds, followed by analysis of the dissociation curve of the amplicon, at temperatures ranging from 65ºC to 95ºC, with 0.5º C increments and reading for five seconds. As positive and negative controls, genomic DNA of *C. parvum* and ultrapure water were used, respectively.

### Analytical sensitivity

*Cryptosporidium baileyi* oocysts isolated from chickens purchased at a live bird market in the city of Araçatuba, state of São Paulo, Brazil, were used to determine the analytical sensitivity of one-tube nested real-time PCR.

Oocysts were propagated by oral inoculation in two-day-old broiler chicks. Fecal samples were collected from day 10 to 13 after inoculation, strained through disposable sieves and stored in 2.5% potassium bichromate at 4ºC (Meireles & Figueiredo, 1992). The oocysts were purified by centrifugal flotation in Sheather’s solution and cesium chloride solution (Arrowood & Donaldson, 1996), followed by incubation in 2% sodium hypochlorite solution for 10 minutes at 4º C, and five steps of dilution in PBS 7.4 and centrifugation at 12,000 g for five minutes to remove residual sodium hypochlorite. Purified oocysts were resuspended in PBS pH 7.4 and stored at 4°C.

### Analytical sensitivity in stool samples containing added oocysts

The analytical sensitivity of one-tube nested real-time PCR was determined using DNA extracted from *C. baileyi* oocysts, which were added to a fecal sample of a chicken negative for *Cryptosporidium* spp. (by microscopy and conventional nested PCR), in the amount of 10^5^ to 10^0^ per gram of feces. The oocysts were quantified in a Neubauer chamber.

DNA extraction was performed using the ZR Fecal DNA MiniPrep™ kit (Zymo Research), according to the manufacturer’s protocol. The reactions were performed in quintuplicate.

### Analytical sensitivity in DNA extracted from purified oocysts

Analytical sensitivity of one-tube nested real-time PCR was determined from DNA extracted from dilutions containing 10^5^ to 10^0^ *C. baileyi* oocysts quantified in a Neubauer chamber.

DNA was extracted using oocysts diluted in a Chelex 100™ solution (Bio-Rad) and then frozen in five steps in liquid nitrogen for one minute and thawed for three minutes at 99º C in a thermomixer (Di Giovanni & LeChevallier, 2005). Reactions were performed in quintuplicate.

### Analytical specificity

The analytical specificity of one-tube nested real-time PCR was determined using genomic DNA from microorganisms that could potentially cross-react with *Cryptosporidium* DNA, such as *Candida* sp., *Isospora* sp. from the lesser seed finch, and several species of *Eimeria* that infect domestic fowl. Reactions were performed in duplicate.

### Repeatability and reproducibility

The repeatability of one-tube nested real-time PCR was determined using 30 avian DNA samples previously diagnosed as positive by one-tube nested real-time PCR, which were tested three times by the same person under the same conditions. To assess the reproducibility of the assay, 30 samples were examined by three different people, all with experience in real-time PCR, in two laboratories, using a CFX96™ Real-Time PCR Detection System (Bio-Rad) and Mastercycler RealPlex™ (Eppendorf).

### Diagnostic sensitivity and specificity

To determine the diagnostic sensitivity and specificity of one-tube nested real-time PCR, genomic DNA samples extracted from individual fecal samples from 443 birds of the orders Psittaciformes, Passeriformes and Galliformes (Table 2) were analyzed by one-tube nested real-time PCR and by conventional nested PCR for amplification of 18S rRNA gene fragments (Xiao et al., 2000), the latter used as the gold standard for the detection of *Cryptosporidium* spp. These DNA samples, which originated from previous studies (Nakamura et al., 2009; Camargo et al., 2018; Ferrari et al., 2018; Santana et al., 2018), had been stored at -20º C in the Laboratory of Avian Pathology at the Faculty of Veterinary Medicine of São Paulo State University – UNESP.

**Table 2:** Results of one-tube real-time nested PCR and conventional nested PCR in avian fecal samples.

**Host**	**No. positive/No. Sampled (% positive)**	**Detection of *Cryptosporidium* spp.**		**Genetic sequencing**	
		**Conventional nested PCR**	**One-tube nested real-time PCR**	**Conventional nested PCR**	**One-tube nested real-time PCR**
Psittaciformes	15/109 (13.8)	-	+	-	*C. proventriculi* 13/15 (86.7%)
					*C. galli* 2/15 (13.3%)
	2/109 (2)	+	+	*C. proventriculi* 2/2 (100%)	*C. proventriculi* 2/2 (100%)
Passeriformes	40/253 (16)	-	+	-	*C. baileyi* 7/40 (17.5%)
					*C. galli* 31/40 (77.5%)
					Avian genotype I 2/40 (5%)
	17/253 (6.7)	+	+	*C. galli* 6/17 (35.3%)	*C. galli* 17/17 (100%)
				nd 11/17 (64.7%)	
	14/253 (5.5)	+	-	nd	-
*Gallus domesticus*	12/81 (14.8)	-	+	-	*C. baileyi* 12/12 (100%)
	3/81 (3.7)	+	+	*C. baileyi* 2/3 (66.7%)	*C. baileyi* 3/3 (100%)
	1/81 (1.2)	-	+	-	*C. meleagridis* 1/1 (100%)
**Total**	**104/443 (23.5)**	**36/443 (8.1)**	**90/443 (20.3)**		

nd: DNA amplified in insufficient amount for sequencing.

Samples subjected to one-tube nested real-time PCR were examined by agarose gel electrophoresis to check for fragments of sizes different from those expected for *Cryptosporidium* spp.

### Conventional nested PCR for amplification of the 18S rRNA gene

Amplification of a fragment of the 18S rRNA gene was performed using the PCR primers 5’-TTCTAGAGCTAATACATGCG-3’ and 5’- CCCATTTCCTTCGAAACAGGA-3’ (1325 bp), and the nested PCR primers 5’-GGAAGGGTTGTATTTATTAGATAAAG-3’ and 5’- AAGGAGTAAGGAACAACCTCCA-3’ (826-840 bp) (Xiao et al., 2000). Each reaction consisted of 22.5 μl of Platinum™ PCR SuperMix (Life Technologies), 2.5 mM MgCl2, 200 nM of each primer, 2.0 μl of target DNA, and 0.6 μg/μl of non-acetylated bovine serum albumin (Sigma-Aldrich). Samples were subjected to initial DNA denaturation at 94º C for 2 minutes, followed by 35 cycles, each consisting of 45 s of denaturation at 94º C, 45 s of annealing at 55º C and 60 s of extension at 72º C, with a final extension of 7 min at 72º C.

Genomic DNA from *C. parvum* and ultrapure water were used as positive and negative controls, respectively.

Samples were subjected to 1.5% agarose gel electrophoresis stained with GelRed™ (Biotium).

### Sequencing of fragments amplified by one-tube nested real-time PCR and by conventional nested PCR

One-tube nested real-time PCR and conventional nested PCR amplicons were purified using an ExoSAP-IT™ PCR Product Cleanup Reagent (Termofisher Scientific).

The amplified fragments were sequenced using an ABI Prism™ Dye Terminator 3.1 kit in an automatic sequencer at the Center for Biological Resources and Genomic Biology (CREBIO) at UNESP, Campus of Jaboticabal. Sequencing reactions were performed in both directions, using primers from the second steps of one-tube nested real-time PCR and conventional nested PCR.

Consensus sequences were determined using CodonCode Aligner version 9.0.1 software (CodonCode Corporation), considering only nucleotides with quality above 20, aligned with the aid of the Clustal W (Thompson et al., 1997) and BioEdit Sequence Alignment Editor (Hall, 1999), and compared with homologous sequences available in GenBank, using the Basic Local Alignment Search Tool – BLAST.

## Results

In the first step of one-tube nested real-time PCR, the best result was achieved when the annealing and extension steps were performed simultaneously, at a temperature of 70°C. In the second step, the annealing temperature of 62°C associated with the extension at 72°C resulted in the best amplification. Among all the combinations of primer concentrations, the best concentrations were 50nM and 400nM in the 1^st^ and 2^nd^ steps, respectively, for both sets of primers of each step.

**Figure 1:**
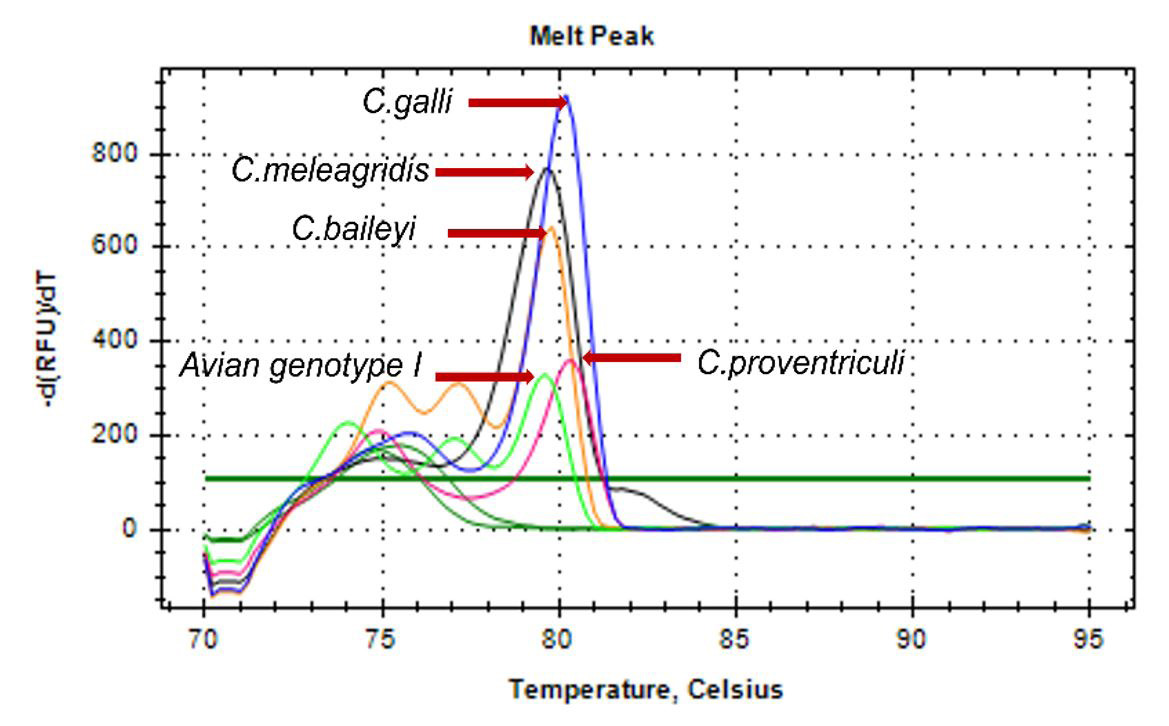
Melting temperature of one-tube nested real-time PCR amplicons of selected avian *Cryptosporidium* species and genotypes: 79.4°C, 79.6°C, 79.6°C, 80°C, and 80.2°C, for avian genotype I, *C. baileyi*, *C. meleagridis, C. galli*, and *C. proventriculi*, respectively.

**Figure 2:**
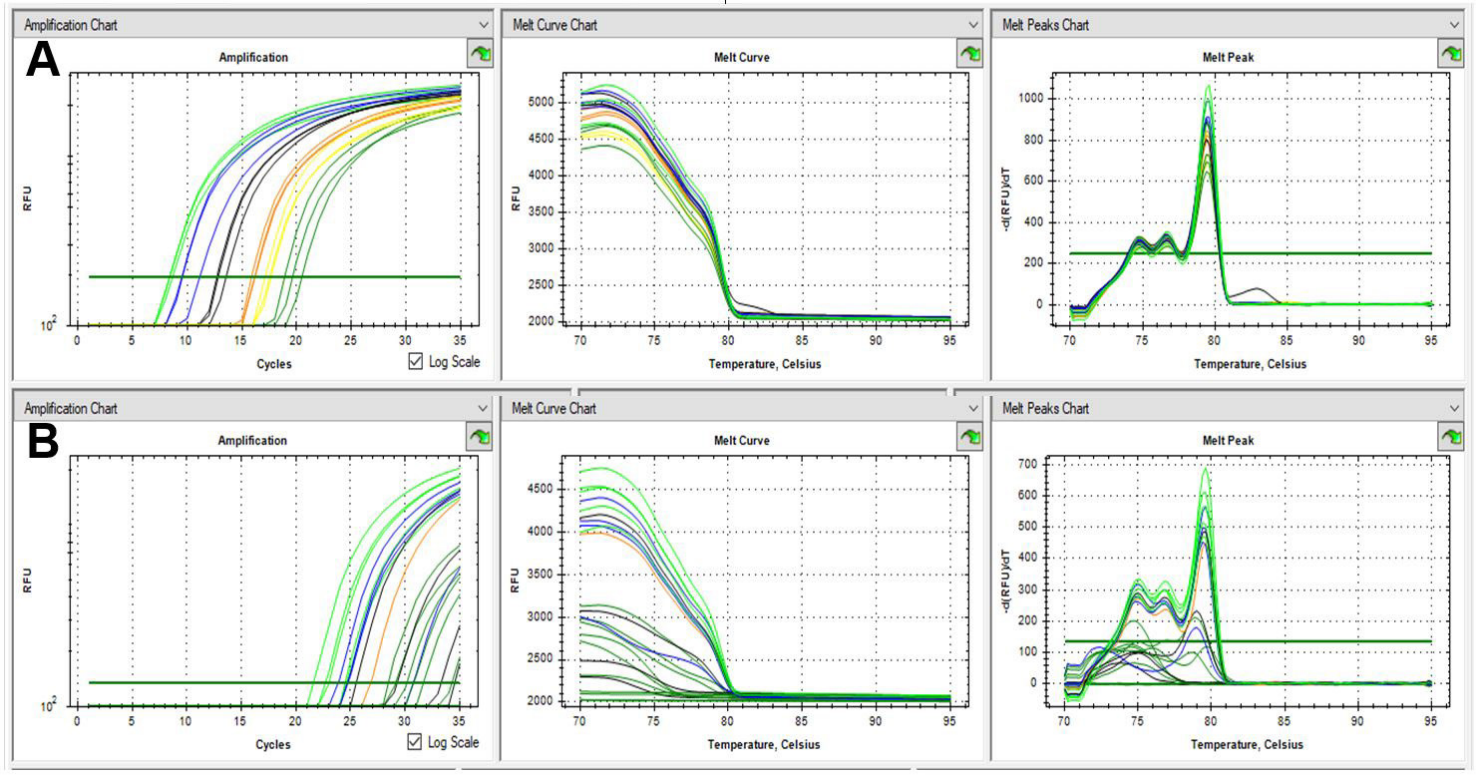
Analytical sensitivity of one-tube nested real-time PCR. (A) DNA extracted from *C. baileyi* purified oocysts; (B) DNA extracted from fecal samples added with *C. baileyi* oocysts. 10^5^ oocysts (light green), 10^4^ oocysts (blue), 10^3^ oocysts (black), 10^2^ oocysts (orange), 10^1^ oocysts (yellow), and 10^0^ oocysts (dark green).

**Figure 3:**
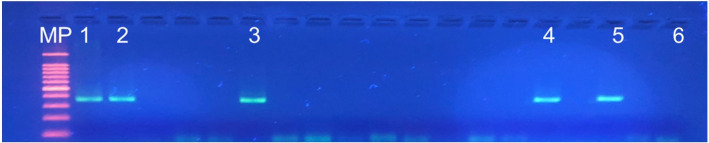
Agarose gel electrophoresis of one-tube nested real-time PCR amplicons from avian fecal samples. Positive control: *C. parvum* (1); *C. baileyi* (2); avian genotype I (3); *C. proventriculi* (4); *C. galli* (5), and negative control (6). Lanes not numbered: negative avian fecal samples. MP: 100 bp DNA ladder marker.

Dissociation temperatures of 79.4°C, 79.6°C, 79.6°C, 80.0°C, and 80.2°C were observed for *Cryptosporidium* avian genotype I, *C. baileyi*, *C. meleagridis*, *C. galli*, and *C. proventriculi*, respectively (Figure 1). An analysis of one-tube nested real-time PCR melting peaks (Figures 1, 2) revealed the presence of double peaks, which correspond to regions in the amplified fragments that present dissociation at different temperatures (Dwight et al., 2011) and do not characterize nonspecific amplification, as evidenced by agarose gel electrophoresis (Figure 3).

One-tube nested real-time PCR detected approximately 0.5 oocyst (2 sporozoites) per reaction. There was no DNA amplification from microorganisms that could potentially cross-react with the primers used for the diagnosis of *Cryptosporidium* spp., including *Candida* sp., *Isospora* sp., and several species of *Eimeria*.

Repeatability, which was calculated by the same person three times in the same device using DNA from avian fecal samples, showed the same result in 27 out of 30 samples (90%). With regard to the reproducibility of one-tube nested real-time PCR, the analysis of 30 samples examined by three different people, all with experience in real-time PCR, in two laboratories and using different devices, showed the same result in 24 samples out of 30 examined (80%).

By conventional nested PCR, 8.1% (36/443) of the samples showed positivity for *Cryptosporidium* spp. In contrast, samples examined by one-tube nested real-time PCR showed 20.3% (90/443) positivity (Table 2).

All the 90 samples amplified by one-tube real-time nested PCR were successfully sequenced, leading to the identification of *C. baileyi, C. galli, C. meleagridis, C. proventriculi*, and *Cryptosporidium* avian genotype I. Genetic sequencing of amplicons from conventional nested PCR identified *Cryptosporidium* species in 10/36 (27.8%) samples, with identification of the same species identified by one-tube nested real-time PCR amplicon sequencing (Table 2).

## Discussion

One-tube nested real-time PCR serves to amplify a fragment of the 18S rRNA gene from *Cryptosporidium* spp. in two steps and in a single tube. The DNA amplified in the first step was used as a template for amplification in the second step, using internal primers, with real-time visualization of the amplification.

One-tube nested real-time PCR showed higher sensitivity than conventional nested PCR, which is considered the gold standard for the detection of *Cryptosporidium* spp. Higher sensitivity of real-time PCR protocols than that of conventional nested PCR has already been reported by several authors (De Waele et al., 2011; Homem et al., 2012; Yang et al., 2013; Nakamura et al., 2014; Silva et al., 2014).

In this study, most samples had amplification at high CT values (~30), probably due to the small amount of DNA present in the samples. Yang et al. (2013) also found positivity for *Cryptosporidium* spp. by real-time PCR only with amplification at high CT values, in samples where there was no detection by conventional nested PCR. This same correlation between high CT values ​​and small amounts of the target microorganism in fecal samples was observed by Fillaux et al. (2008), using real-time PCR for the diagnosis of *Pneumocystis jirovecii* in humans.

For validation of a diagnostic protocol, the diagnostic specificity and sensitivity and the positive and negative predictive values ​​of the reaction must also be determined. The sensitivity of one-tube nested real-time PCR was higher than that of conventional nested PCR (gold standard), thus precluding these calculations. This is because the result would indicate low specificity of one-tube nested real-time PCR, given that all the results related to samples positive by one-tube nested real-time PCR and negative by conventional nested PCR would be considered false positives, as has been observed in other studies involving the validation of real-time PCR protocols (De Waele et al., 2011; Homem et al., 2012; Nakamura et al., 2014; Silva et al., 2014). The possibility of false-positive results was ruled out by sequencing the fragments amplified by one-tube nested real-time PCR, which proved that there was no unspecific amplification.

In this study, the viability of sequencing from the product of one-tube nested real-time PCR was determined, without agarose gel electrophoresis and without the need for re-amplification to obtain higher DNA yield. The one-tube nested real-time PCR protocol does not amplify unspecific fragments, so the fragments resulting from this reaction were purified directly from the reaction product using the enzymatic reagent ExoSAP-IT®.

Variations in repeatability and reproducibility results are most likely related to both the variability of the reaction/people/device and the uneven distribution of DNA in the sample (Bustin & Nolan, 2004), which is due to the small amount of DNA present in samples from birds infected by some species of *Cryptosporidium*, particularly gastric species in birds (Nakamura & Meireles, 2015).

The protocol standardized in this study uses primers to amplify a fragment of approximately 432 bp, which is larger than that recommended for real-time PCR protocols. This characteristic of one-tube nested real-time PCR certainly interfered in the efficiency of the reaction (Arya et al., 2005), but even with this limitation, one-tube nested real-time PCR showed higher sensitivity than the gold standard assay. These primers were chosen due to a particularity of the 18S rRNA gene of *Cryptosporidium*, which is the existence of a particular region with a highly conserved nucleotide sequence in all species and genotypes, which contains polymorphic sequences that allow for differentiation between *Cryptosporidium* species and genotypes (Xiao et al., 1999). This region was selected for amplification by one-tube nested real-time PCR, since there was no alternative for the selection of primers that would anneal in another region of the 18S rRNA gene that were genus-specific and enabled the differentiation of species and genotypes by genetic sequencing. In this project, this limitation prevented the optimization of the reaction using primers directed at other regions of the gene.

This study enabled the validation of a diagnostic protocol which is highly sensitive and specific for the detection of *Cryptosporidium* spp. in avian fecal samples, in addition to enabling amplicon purification and sequencing directly from the amplified product. Some of the advantages of this protocol are its performance in a single step, the fact that it does not require electrophoresis to visualize the amplified product, and its high specificity and sensitivity, which are superior to those of conventional nested PCR. These characteristics reduce the possibility of contamination, produce faster results, offer cost savings in reagents, and provide more reliable results.

## Conclusions

One-tube nested real-time PCR assay was validated for the detection of *Cryptosporidium* spp. in avian fecal samples. One-tube nested real-time PCR showed higher sensitivity than the protocol considered the gold standard for the detection of *Cryptosporidium* spp. in avian fecal samples. Genetic sequencing of one-tube nested real-time PCR amplicons can be performed after purification by enzymatic assay protocols, even in samples with high CT values.

## References

[B001] Abe N, Makino I (2010). Multilocus genotypic analysis of *Cryptosporidium* isolates from cockatiels, Japan. Parasitol Res.

[B002] Arrowood MJ, Donaldson K (1996). Improved purification methods for calf-derived *Cryptosporidium parvum* oocysts using discontinuous sucrose and cesium chloride gradients. J Eukaryot Microbiol.

[B003] Arya M, Shergill IS, Williamson M, Gommersall L, Arya N, Patel HRH (2005). Basic principles of real-time quantitative PCR. Expert Rev Mol Diagn.

[B004] Atkinson C, Emery VC, Griffiths PD (2014). Development of a novel single tube nested PCR for enhanced detection of cytomegalovirus DNA from dried blood spots. J Virol Methods.

[B005] Bamaiyi PH, Umoh JU, Abdu PA, Lawal IA (2013). The prevalence of *Cryptosporidium* oocysts in birds in Zaria, Nigeria. Borneo J Resource Sci Technol.

[B006] Blagburn BL, Lindsay DS, Giambrone J, Sundermann CA, Hoerr FJ (1987). Experimental cryptosporidiosis in broiler chickens. Poult Sci.

[B007] Bustin SA, Nolan T (2004). Pitfalls of quantitative real-time reverse-transcription polymerase chain reaction. J Biomol Tech.

[B008] Camargo VDS, Santana BN, Ferrari ED, Nakamura AA, Nagata WB, Nardi ARM (2018). Detection and molecular characterization of *Cryptosporidium* spp. in captive canaries (*Serinus canaria*) using different diagnostic methods. Rev Bras Parasitol Vet.

[B009] Choi Y, Jeon BY, Shim TS, Jin H, Cho SN, Lee H (2014). Development of a highly sensitive one-tube nested real-time PCR for detecting *Mycobacterium tuberculosis.*. Diagn Microbiol Infect Dis.

[B010] Costa J, Oliveira MBPP, Mafra I (2013). Novel approach based on single-tube nested real-time PCR to detect almond allergens in foods. Food Res Int.

[B011] Current WL, Upton SJ, Haynes TB (1986). The life cycle of *Cryptosporidium baileyi* n. sp. (Apicomplexa, Cryptosporidiidae) infecting chickens. J Protozool.

[B012] De Waele V, Berzano M, Berkvens D, Speybroeck N, Lowery C, Mulcahy GM (2011). Age-stratified Bayesian analysis to estimate sensitivity and specificity of four diagnostic tests for detection of *Cryptosporidium* oocysts in neonatal calves. J Clin Microbiol.

[B013] Di Giovanni GD, LeChevallier MW (2005). Quantitative-PCR assessment of *Cryptosporidium parvum* cell culture infection. Appl Environ Microbiol.

[B014] Dwight Z, Palais R, Wittwer CT (2011). uMELT: prediction of high-resolution melting curves and dynamic melting profiles of PCR products in a rich web application. Bioinformatics.

[B015] Eladl AH, Hamed HR, Khalil MR (2014). Consequence of *Cryptosporidiosis* on the immune response of vaccinated broiler chickens against Newcastle disease and/or avian influenza. Vet Res Commun.

[B016] Ewald MPC, Martins FDC, Caldart ET, Vieira FEG, Yamamura MH, Sasse JP (2017). The first study of molecular prevalence and species characterization of *Cryptosporidium* in free-range chicken (*Gallus gallus domesticus*) from Brazil. Rev Bras Parasitol Vet.

[B017] Ferrari ED, Nakamura AA, Nardi ARM, Santana BN, da Silva Camargo V, Nagata WB (2018). *Cryptosporidium* spp. in caged exotic psittacines from Brazil: evaluation of diagnostic methods and molecular characterization. Exp Parasitol.

[B018] Fillaux J, Malvy S, Alvarez M, Fabre R, Cassaing S, Marchou B (2008). Accuracy of a routine real-time PCR assay for the diagnosis of *Pneumocystis jirovecii* pneumonia. J Microbiol Methods.

[B019] Gong Z, Kan ZZ, Huang JM, Fang Z, Liu XC, Gu YF (2021). Molecular prevalence and characterization of *Cryptosporidium* in domestic free-range poultry in Anhui Province, China. Parasitol Res.

[B020] Hadfield S, Robinson G, Elwin K, Chalmers RM (2011). Detection and differentiation of *Cryptosporidium* spp. in human clinical samples by use of real-time PCR. J Clin Microbiol.

[B021] Hall TA (1999). BioEdit: a user-friendly biological sequence alignment editor and analysis program for Windows 95/98/NT. Nucleic Acids Symp Ser.

[B022] Helmy YA, Krücken J, Abdelwhab ESM, von Samson-Himmelstjerna GV, Hafez HM (2017). Molecular diagnosis and characterization of *Cryptosporidium* spp. in turkeys and chickens in Germany reveals evidence for previously undetected parasite species. PLoS One.

[B023] Hoerr FJ, Current WL, Haynes TB (1986). Fatal cryptosporidiosis in quail. Avian Dis.

[B024] Holubová N, Sak B, Horčičková M, Hlásková L, Květoňová D, Menchaca S (2016). *Cryptosporidium avium* n. sp. (Apicomplexa: Cryptosporidiidae) in birds. Parasitol Res.

[B025] Holubová N, Tůmová L, Sak B, Hejzlarová A, Konečný R, McEvoy J (2020). Description of *Cryptosporidium ornithophilus* n. sp. (Apicomplexa: Cryptosporidiidae) in farmed ostriches. Parasit Vectors.

[B026] Holubová N, Zikmundová V, Limpouchová Z, Sak B, Konečný R, Hlásková L (2019). *Cryptosporidium proventriculi* sp. n. (Apicomplexa: Cryptosporidiidae) in Psittaciformes birds. Eur J Protistol.

[B027] Homem CG, Nakamura AA, Silva DC, Teixeira WFP, Coelho WMD, Meireles MV (2012). Real-time PCR assay targeting the actin gene for the detection of *Cryptosporidium parvum* in calf fecal samples. Parasitol Res.

[B028] Johnson DW, Pieniazek NJ, Griffin DW, Misener L, Rose JB (1995). Development of a PCR protocol for sensitive detection of *Cryptosporidium* oocysts in water samples. Appl Environ Microbiol.

[B029] Liu X, Zhu H, Meng W, Dong H, Han Q, An Z (2019). Occurrence of a *Cryptosporidium xiaoi*–like genotype in peafowl (*Pavo cristatus*) in China. Parasitol Res.

[B030] Makino I, Abe N, Reavill DR (2010). *Cryptosporidium* avian genotype III as a possible causative agent of chronic vomiting in peach-faced lovebirds (*Agapornis roseicollis*). Avian Dis.

[B031] Meireles MV, Figueiredo PC (1992). 1996 (Apicomplexa: Cryptosporidiidae) em frangos de corte. Rev Bras Parasitol Vet.

[B032] Meireles MV, Soares RM, dos Santos MM, Gennari SM (2006). Biological studies and molecular characterization of a *Cryptosporidium* isolate from ostriches (*Struthio camelus*). J Parasitol.

[B033] Minarovicová J, Kaclíková E, Krascsenicsová K, Siekel P, Kuchta T (2009). A single-tube nested real-time polymerase chain reaction for sensitive contained detection of *Cryptosporidium parvum.*. Lett Appl Microbiol.

[B034] Naciri M, Mazzella O, Coudert F (1989). Interactions cryptosporidies-virus sauvage ou vaccinal de la maladie de Marek chez le poulet. Rec Méd Vét.

[B035] Nakamura AA, Homem CG, Da Silva AMJ, Meireles MV (2014). Diagnosis of gastric cryptosporidiosis in birds using a duplex real-time PCR assay. Vet Parasitol.

[B036] Nakamura AA, Meireles MV (2015). *Cryptosporidium* infections in birds - a review. Rev Bras Parasitol Vet.

[B037] Nakamura AA, Simões DC, Antunes RG, Da Silva DC, Meireles MV (2009). Molecular characterization of *Cryptosporidium* spp. from fecal samples of birds kept in captivity in Brazil. Vet Parasitol.

[B038] Ng J, Pavlasek I, Ryan U (2006). Identification of novel *Cryptosporidium* genotypes from avian hosts. Appl Environ Microbiol.

[B039] Qi M, Wang R, Ning C, Li X, Zhang L, Jian F (2011). *Cryptosporidium* spp. in pet birds: genetic diversity and potential public health significance. Exp Parasitol.

[B040] Ravich ML, Reavill DR, Hess L, Childress AL, Wellehan JFX (2014). Gastrointestinal cryptosporidiosis in captive psittacine birds in the United States: A case review. J Avian Med Surg.

[B041] Ryan UM, Xiao L, Read C, Sulaiman IM, Monis P, Lal AA (2003). A redescription of *Cryptosporidium galli* Pavlásek, 1999 (Apicomplexa: Cryptosporidiidae) from birds. J Parasitol.

[B042] Santana BN, Kurahara B, Nakamura AA, da Silva Camargo V, Ferrari ED, da Silva GS (2018). Detection and characterization of *Cryptosporidium* species and genotypes in three chicken production systems in Brazil using different molecular diagnosis protocols. Prev Vet Med.

[B043] Santos MMAB, Peiró JR, Meireles MV (2005). *Cryptosporidium* infection in ostriches (*Struthio camelus*) in Brazil: clinical, morphological and molecular studies. Braz J Poult Sci.

[B044] Shen XX, Qiu FZ, Zhao HL, Yang MJ, Hong L, Xu ST (2018). A novel and highly sensitive real-time nested RT-PCR assay in a single closed tube for detection of enterovirus. Diagn Microbiol Infect Dis.

[B045] Silva DC, Paiva PRSO, Nakamura AA, Homem CG, Souza CG, Grego KF (2014). The detection of *Cryptosporidium serpentis* in snake fecal samples by real-time PCR. Vet Parasitol.

[B046] Slavin D. (1955). *Cryptosporidium meleagridis* (Sp. Nov.). J Comp Pathol.

[B047] Sréter T, Varga I (2000). Cryptosporidiosis in birds – a review. Vet Parasitol.

[B048] Staggs SE, Beckman EM, Keely SP, Mackwan R, Ware MW, Moyer AP (2013). The applicability of TaqMan-based quantitative real-time PCR assays for detecting and enumerating *Cryptosporidium* spp. oocysts in the environment. PLoS One.

[B049] Sun Y, Chen J, Li J, Xu Y, Jin H, Xu N (2017). Novel approach based on one-tube nested PCR and a lateral flow strip for highly sensitive diagnosis of tuberculous meningitis. PLoS One.

[B050] Thompson JD, Gibson TJ, Plewniak F, Jeanmougin F, Higgins DG (1997). The CLUSTAL_X windows interface: flexible strategies for multiple sequence alignment aided by quality analysis tools. Nucleic Acids Res.

[B051] Thongsri Y, Wonglakorn L, Chaiprasert A, Svobodova L, Hamal P, Pakarasang M (2013). Evaluation for the clinical diagnosis of *Pythium insidiosum* using a single-tube nested PCR. Mycopathologia.

[B052] Toohey-Kurth K, Reising MM, Tallmadge RL, Goodman LB, Bai J, Bolin SR (2020). Suggested guidelines for validation of real-time PCR assays in veterinary diagnostic laboratories. J Vet Diagn Invest.

[B053] Xiao L, Alderisio K, Limor J, Royer M, Lal AA (2000). Identification of species and sources of *Cryptosporidium* oocysts in storm waters with a small-subunit rRNA-based diagnostic and genotyping tool. Appl Environ Microbiol.

[B054] Xiao L, Escalante L, Yang C, Sulaiman I, Escalante AA, Montali RJ (1999). Phylogenetic Analysis of *Cryptosporidium* parasites based on the small-subunit rRNA gene locus. Appl Environ Microbiol.

[B055] Yang R, Murphy C, Song Y, Ng-Hublin J, Estcourt A, Hijjawi N (2013). Specific and quantitative detection and identification of *Cryptosporidium hominis* and *C. parvum* in clinical and environmental samples. Exp Parasitol.

[B056] Zylan K, Bailey T, Smith HV, Silvanose C, Kinne J, Schuster RK (2008). An outbreak of cryptosporidiosis in a collection of Stone curlews (*Burhinus oedicnemus*) in Dubai. Avian Pathol.

